# Supplementary material for: Microscopic study of the Halperin–Laughlin interface through matrix product states
Source: Nat Commun. 2019 Apr 23;10:1860. doi: 10.1038/s41467-019-09169-y (PMC6478930; doi:10.1038/s41467-019-09169-y)
Supplement: Supplementary file 1 — Supplementary Information [file 41467_2019_9169_MOESM1_ESM.pdf]

Supplementary Information: Microscopic Study of the Halperin - Laughlin Interface through Matrix Product States

V. Crépel[1], N. Claussen[1], N. Regnault[1] and B. Estienne[2]
[1]*Laboratoire de Physique de l'École Normale supérieure, ENS, Université PSL, CNRS, Sorbonne Université, Université Paris Diderot, Sorbonne Paris Cité, Paris, France and*
[2]*Sorbonne Université, CNRS, Laboratoire de Physique Théorique et Hautes Énergies, LPTHE, F-75005 Paris, France*

Supplementary Tab. I. *Right and left MPS boundary charges $(n_{\rm L}, n_\perp)$ to recover the glued Laughlin 1/2 and Halperin 221 root partitions. $\emptyset$, $\downarrow$ and $\uparrow$ respectively denote an empty orbitals or an occupied orbital with a spin down or up. Using the first and the second columns, the total root configuration should be understood as ...$\emptyset \downarrow \emptyset \downarrow - \emptyset \uparrow\downarrow \emptyset \uparrow\downarrow$... for $[\emptyset \downarrow]$ and $[\emptyset \uparrow\downarrow]$ (e.g. the case shown in the first row). Note that defining the root configuration by, e.g., $[\emptyset \uparrow\downarrow]$ instead of $[\emptyset \downarrow\uparrow]$ is arbitrary due to the SU(2) singlet nature of the Halperin 221 state.*

Root Partition Laughlin	Root Partition Halperin	$(n_{\rm L}, n_\perp)$
$[\emptyset \downarrow]$	$[\emptyset \uparrow\downarrow]$	$(0,0)$
$[\emptyset \downarrow]$	$[\uparrow \emptyset \downarrow]$	$(0,2)$
$[\emptyset \downarrow]$	$[\uparrow\downarrow \emptyset]$	$(0,4)$
$[\downarrow \emptyset]$	$[\emptyset \uparrow\downarrow]$	$(1,-3)$
$[\downarrow \emptyset]$	$[\uparrow \emptyset \downarrow]$	$(1,-1)$
$[\downarrow \emptyset]$	$[\uparrow\downarrow \emptyset]$	$(1,1)$

Supplementary Tab. II. *Overlap between the MPS variational ansatz for the $(n_{\rm L}, n_\perp) = (0,0)$ and $P_\mu = P_\eta = 0$ boundary conditions and the corresponding ED ground state for different system sizes characterized by the particle numbers $(2N_{\rm H}, N_{\rm L})$. The number of orbitals are fixed to $N_{\rm orb}^{\rm L} = 2N_{\rm L} - 1$ and $N_{\rm orb}^{\rm H} = 3N_{\rm H}$. Due to the dimension of the many-body Hilbert considered (222 415 944 for the largest systems), the overlaps are computed over a significant fraction of the vectors weights. The norms of the truncated ED $|\psi_{\rm trunc}^{\rm ED}\rangle$ and MPS $|\psi_{\rm trunc}^{\rm MPS}\rangle$ vectors, which can be evaluated rigorously, give an estimate for the possible error.*

$(2N_{\rm H}, N_{\rm L})$	$\|\| \, \|\psi_{\rm trunc}^{\rm ED}\rangle \, \|\|$	$\|\| \, \|\psi_{\rm trunc}^{\rm MPS}\rangle \, \|\|$	Overlap $\|\langle\psi_{\rm trunc}^{\rm ED}\|\psi_{\rm trunc}^{\rm MPS}\rangle\|$
(6,3)	1.00000	1.00000	0.99961
(6,4)	1.00000	1.00000	0.99894
(8,5)	0.99997	0.99997	0.99891

Supplementary Note I. Comparison with Exact Diagonalization - Bosonic Case

We have also put the bosonic model ($m = 2$) discussed in Ref.[1] under scrutiny of finite size ED. The results that we have obtained underline the microscopic validity of the model state and its applicability to both fermions and bosons. For the bosonic case, we rely on the following interacting Hamiltonian

$$\mathcal{H} = \int {\rm d}^2\mathbf{r} \sum_{\sigma,\sigma' \in \{\uparrow,\downarrow\}} : \rho_\sigma(\mathbf{r})\rho_{\sigma'}(\mathbf{r}) + \mu_\uparrow \rho_\uparrow(\mathbf{r}) \,, \tag{1}$$

which once projected to the LLL admits the Laughlin 1/2 (resp. Halperin 221) state as its densest zero energy state when $\mu_\uparrow \to \infty$ (resp. $\mu_\uparrow = 0$). The electronic operators $\mathcal{W}^\uparrow$ and $\mathcal{W}^\downarrow$ generate the charge lattice $\frac{n_\perp + (m-1)n_{\rm L}}{m} \in \mathbb{Z}$ from a unit cell composed of 3 inequivalent sites[2]. Physically, they correspond to the ground state degeneracy of the Halperin 221 state on the torus (or infinite cylinder) which is known to be $|\det \mathbf{K}| = 3^3$. Hence, the choice of $n_\perp$ modulo three determines the topological sector of the Halperin bulk far from the transition. An identical analysis involving the spin down electronic operator $\mathcal{W}^\downarrow$ alone shows that the Laughlin topological sector is selected by the parity of $n_{\rm L}$ far from the transition on the polarized phase. The discussion about the gluing of root partition may be reproduced and the possible choices are summarized in Supplementary Tab. I. As in the main text, the choice of the U(1)-charges selects a root configuration of Supplementary Tab. I, which fixes the reference for the total angular momentum of the system K_y in finite size. Low energy excitations are obtained by dialing the $\varphi^{\rm L}$ and $\varphi^\perp$ descendants.

Using the same ED procedure as in the main text for the Hamiltonian of Supplementary Eq. (1), we have localized the states which persist in the thin cylinder limit $L \ll \ell_B$. We observe that, when $N_{\rm orb}^{\rm L} = 2N_{\rm L} - 1$ and $N_{\rm orb}^{\rm H} = 3N_{\rm H}$, the ED ground state has a total momentum equal to the one selected by the $(n_{\rm L}, n_\perp) = (0,0)$ boundary charges (see Supplementary Tab. I and the $N_{\rm orb}^{\rm L} = 5$ case in Supplementary Fig. 1). Our MPS ansatz with these boundary conditions shows extremely high overlap with the corresponding ED ground states (see Supplementary Tab. II). While the ED spectrum does not distinguish between the Laughlin and Halperin bulk excitations and the excitations of interface modes, the high overlap between the MPS states with the low lying part of the ED spectra help us discriminating these different types of excitations. To illustrate this, we will first look at the role of the level descendant P_R and P_L when the boundary U(1)-charges are fixed to $(n_{\rm L}, n_\perp) = (0,0)$ and then consider the other gluing conditions of Supplementary Tab. I. As in the main text, we consider a system of $(2N_{\rm H}, N_{\rm L}) = (6,3)$ particles in $N_{\rm orb}^{\rm L} = 2N_{\rm L} - 1$ and $N_{\rm orb}^{\rm H} = 3N_{\rm H}$ orbitals (orange spectrum in Supplementary Fig. 1). As stated earlier, the choice in boundary charges $(n_{\rm L}, n_\perp) = (0,0)$ fixes the reference of momentum by selecting a specific root configuration and the ground

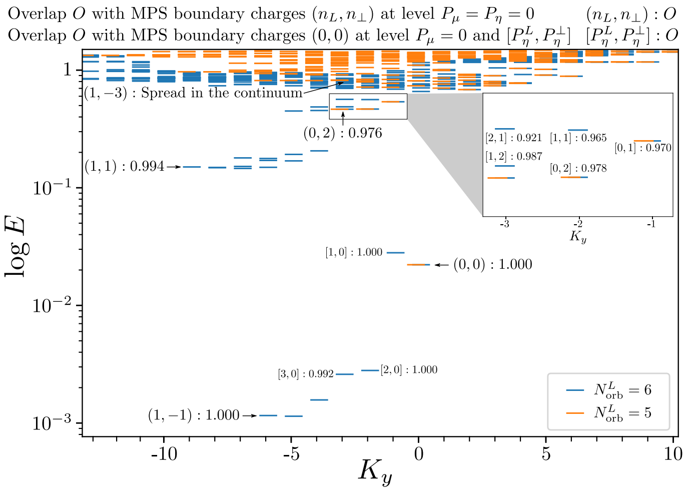

Supplementary Fig. 1. **Test of the bosonic ansatz:** Energy spectrum (using a logarithmic scale) of Supplementary Eq. (1) for a system of 6 particles (6 spin down, 3 spin up) on a cylinder of perimeter $L = 9\ell_B$ with $N_{\rm orb}^{\rm H} = 9$ orbitals and $N_{\rm orb}^{\rm L} = 5$ (orange) or $N_{\rm orb}^{\rm L} = 6$ (blue). In the former case, only one of the root configuration of Supplementary Tab. I can be produced and we can follow interface excitations exactly ($P_L^- = 0$ in square bracket symbols – see text). When $N_{\rm orb}^{\rm L} = 6$, excitations of the Laughlin bulk may arise and other root configurations are also possible and are shown with round bracket symbols, together with the overlap with the ED states targeted. We have also indicated in square bracket some excitations for an MPS with boundary charges $(0,0)$ which reproduce faithfully some of the ED states at low energy. The other low lying states may be reproduced with the same kind of excitations onto MPS with boundary charges $(1,-1)$, $(1,1)$, $(0,2)$ and $(0,4)$. In the later two cases, we should also consider excitations on the Halperin side, *i.e.* $P_R \neq 0$. Overall, we were able to faithfully capture all the low energy ($E < 0.65$) features of the spectrum with overlaps ranging from 0.921 to 1.000.

state corresponds to the case $P_\mu - P_\eta = 0$. More generally, the MPS model WF has total momentum $P_\mu - P_\eta$ with respect to the one of the chosen root configuration. We furthermore fix $P_\mu = 0$ and label as $[P_\eta^L, P_\eta^\perp]$ (using square brackets) the subspaces generated by the corresponding WFs. The lowest energy states detaching from the continuum are found to be well captured by the MPS states with boundary condition $P_\eta^\perp$ (see the overlaps $[0, P_\eta^\perp]$ in Supplementary Fig. 1). Contrary to bulk excitations, their energies barely change when orbitals are added to the side of the system and they are found to be localized at the interface.

Adding an extra orbital on the Laughlin side allows to probe low energy excitations at the edge of the Laughlin bulk. They are characterized by their level descendant P_η^L and capture extremely well the lowest energy states appearing for $N_{\rm orb}^{\rm L} = 6$ (see the overlaps $[P_\eta^L, 0]$ in Supplementary Fig. 1). Moreover, the root partition dominating the low energy feature of the spectrum might change, as in finite size such change only requires a finite momentum transfer. Changing the charge sector as prescribed in Supplementary Tab. I, we could discriminate between the different gluing of root partitions. The remaining low lying states may be reproduced with the same kind of excitations $[P_\eta^L, P_\eta^\perp]$ onto MPS with boundary charges $(1,-1) - (1,1) - (0,2)$ and $(0,4)$. In the later two cases, one should also consider excitations on the Halperin side, *i.e.* $P_\mu \neq 0$. Overall, we were able to faithfully capture all the low energy ($E < 0.65$) features of the spectrum with overlaps ranging from 0.921 to 1.000 (as a rule of thumb, the closer to the continuum the poorer the MPS ansatz performs).

Supplementary Note II. Beyond Finite Size: Dispersion Relation of the Critical Mode

Following Refs.[4,5], we expressed the Hamiltonian of Supplementary Eq. (1) as a Matrix Product Operator (MPO) in order to overcome finite-size effects of ED. For such long range Hamiltonians[6,7], exact MPO representations involve an infinite bond dimension $\chi_{\rm MPO}$ and practical implementations require to approximate the MPO's action on an MPS. An efficient and memory effective way to do so, which dramatically decreases $\chi_{\rm MPO}$ compared to other methods[5], is to model the Hamiltonian by a sum of exponentials[8,9] which possess each a rank 3 MPO representation. Keeping up to 8 exponential terms, we obtained an MPO of bond dimension $\chi_{\rm MPO} = 936$ which faithfully describe the Hamiltonian of Supplementary Eq. (1) for the perimeters considered. A major advantage of our approach is the ability to focus solely on the edge mode at the interface. Indeed, ED would show the combination of all possible excitations as discussed previously, scrambling the dispersion relation of the gapless interface mode. In Supplementary Fig. 2, we show the dispersion relation of the critical interface mode computed for a system of 60 orbitals (we have checked the

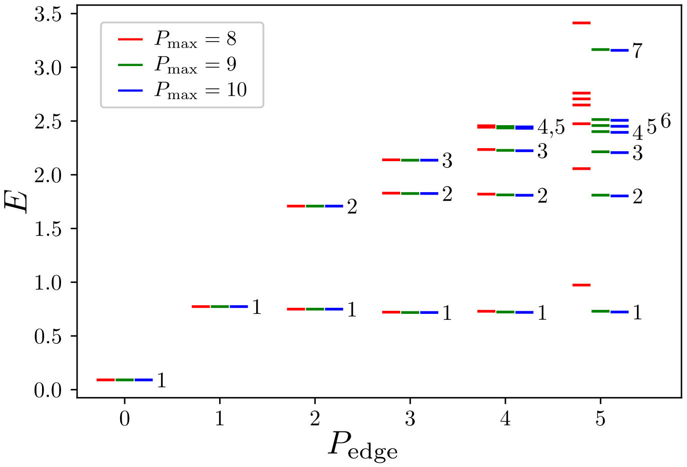

Supplementary Fig. 2. **Dispersion relation beyond finite size:** Energy of the MPS ansatz when momentum excitations of the critical interface mode are controlled via the MPS boundary level descendant $P_{\rm edge}$ on the Laughlin side. The system consists of 60 orbitals (30 on he Laughlin side, 30 on the Halperin side) at perimeter $L = 12\ell_B$. The use of MPO allows to combine a fine control over the excitation provided by our MPS ansatz, and to reach system sizes unreachable with ED.

convergence with the number of orbitals). It exhibits the counting statistics of a massless boson and a linear upper branch, as expected by the theoretical predictions and our results. The mode velocity is extremely high with a large spread per momentum sector. This explains why the excitations $P_{\rm edge} > 2$ reach the continuum. We attribute these energetic behaviors to the sharpness of the transition described by our ansatz, *i.e.* to the change from $\mu_\uparrow = 0$ to $\mu_\uparrow = \infty$ over an inter-orbital distance. Because of the strong and sharp confinement of spin up in the Halperin region, large corrections to this linear behavior are expected due to scattering between the excited modes (see discussion in Ref.[10] for a similar situation). We believe that a smooth transition would provide a more realistic spectrum and decent velocity. A corresponding ansatz could be derived by using a similar approach to the RSES, namely a weighted combination of the two types of matrices.

1 V. Crépel, N. Claussen, B. Estienne, and N. Regnault, arXiv:1806.06858 / Draft NCOMMS-18-20389A.
2 V. Crépel, B. Estienne, B. A. Bernevig, P. Lecheminant, and N. Regnault, Phys. Rev. B **97**, 165136 (2018).
3 X. G. Wen and A. Zee, Phys. Rev. B **46**, 2290 (1992).
4 M. P. Zaletel, R. S. K. Mong, and F. Pollmann, Phys. Rev. Lett. **110**, 236801 (2013).
5 M. P. Zaletel, R. S. K. Mong, F. Pollmann, and E. H. Rezayi, Phys. Rev. B **91**, 045115 (2015).
6 S. A. Trugman and S. Kivelson, Phys. Rev. B **31**, 5280 (1985).
7 F. D. M. Haldane, Phys. Rev. Lett. **51**, 605 (1983).
8 G. M. Crosswhite, A. C. Doherty, and G. Vidal, Phys. Rev. B **78**, 035116 (2008).
9 B. Pirvu, V. Murg, J. I. Cirac, and F. Verstraete, New J. Phys. **12**, 025012 (2010).
10 R. Fern and S. H. Simon, Phys. Rev. B **95**, 201108 (2017).
